# Skin microbiota in frogs from the Brazilian Atlantic Forest: Species, forest type, and potential against pathogens

**DOI:** 10.1371/journal.pone.0179628

**Published:** 2017-07-05

**Authors:** Ananda Brito de Assis, Cristine Chaves Barreto, Carlos Arturo Navas

**Affiliations:** 1 Department of Physiology, Institute of Biosciences, University of São Paulo, São Paulo, São Paulo, Brazil; 2 Graduate Program in Genomic Sciences and Biotechnology, Catholic University of Brasília, Brasília, Brazil; Universitat Trier, GERMANY

## Abstract

The cutaneous microbiota of amphibians can be defined as a biological component of protection, since it can be composed of bacteria that produce antimicrobial compounds. Several factors influence skin microbial structure and it is possible that environmental variations are among one of these factors, perhaps through physical-chemical variations in the skin. This community, therefore, is likely modified in habitats in which some ecophysiological parameters are altered, as in fragmented forests. Our research goal was to compare the skin bacterial community of four anuran species of the Atlantic Forest of Brazil in landscapes from two different environments: continuous forest and fragmented forest. The guiding hypotheses were: 1) microbial communities of anuran skin vary among sympatric frog species of the Atlantic forest; 2) the degree to which forested areas are intact affects the cutaneous bacterial community of amphibians. If the external environment influences the skin microbiota, and if such influences affect microorganisms capable of inhibiting the colonization of pathogens, we expect consequences for the protection of host individuals. We compared bacterial communities based on richness and density of colony forming units; investigated the antimicrobial potential of isolated strains; and did the taxonomic identification of isolated morphotypes. We collected 188 individual frogs belonging to the species *Proceratophrys boiei*, *Dendropsophus minutus*, *Aplastodiscus leucopygius* and *Phyllomedusa distincta*, and isolated 221 bacterial morphotypes. Our results demonstrate variation in the skin microbiota of sympatric amphibians, but only one frog species exhibited differences in the bacterial communities between populations from fragmented and continuous forest. Therefore, the variation we observed is probably derived from both intrinsic aspects of the host amphibian species and extrinsic aspects of the environment occupied by the host. Finally, we detected antimicrobial activity in 27 morphotypes of bacteria isolated from all four amphibian species.

## Introduction

Studies on the skin microbiota of amphibians have increased since the pioneer publication by Bettin and Greven [1], who reported the presence of bacteria on the skin of the urodele *Salamandra salamandra*. This observation was corroborated in other species, including, *Plethodon ventralis*, with the additional observation that metabolites produced by skin bacteria could prevent fungal infection of the eggs cared for by females [2]. It is now clear that microbial communities thrive on the skin of amphibians, and that some bacteria belonging to these communities can inhibit the growth of skin pathogens [3–5]. More recently, bioaugmentation experiments suggest that bacterial species isolated from amphibian skin can be used to inoculate infected animals, and reduce symptoms and mortality caused by the amphibian pathogenic fungus *Batrachochytrium dendrobatidis* (*Bd*) [6], a fungus responsible for population declines of amphibians in different parts of the world [7]. Although it varies widely, the skin microbiota of amphibians is currently perceived as a component of the cutaneous barrier of protection, particularly against disease caused by pathogenic microorganisms of the skin [8–9].

A more complete understanding of the diversity, origin, and role of the amphibian skin microbiota, including the influence of host species and habitat, has become an important issue in the complex web of interactions related to emergent disease in this vertebrate group. Some relevant topics include the composition and role of bacterial communities in the amphibian habitat [10] and the antimicrobial properties of substances produced by skin bacteria [11,12]. Variation in the skin microbial community could be induced by environmental shifts, eventually affecting its potential as an inhibitory mechanism for pathogens. Under the Baas-Becking model, the environment is a selective filter, partially accounting for the spacial variation of microbial diversity [13]. If the skin microbiota of amphibians supports this postulate, the skin bacterial community structure will be largely modulated by the physicochemical microenvironment of the amphibian skin, and less by the biotic interactions occurring within the bacterial community.

Physicochemical characteristics of amphibian skin are likely related to lineage-specific traits, such as the profile of skin secretion components and skin morphology, but also by behavior, mainly in terms of microhabitats and patterns of activity [14,15]. These are likely influenced by temperature, water cycling, pH, and radiation incidence [16] of the environment. An interaction between lineage-specific traits and environment is expected, mainly because environmental changes can affect climate at the individual level of the amphibian hosts, and modify mucus and lipid secretion, two components in the hydrothermal regulation of amphibians [17,18]. Furthermore, changes in the properties of the bioactive molecules with antimicrobial properties, which can act as a negative filter constraining the colonization by some bacterial species [19], also could modulate the structure of the amphibian skin microbiota.

The degree of habitat deforestation is one environmental factor that may influence the microbial communities of amphibian skin. The Atlantic Forest of Brazil is an ideal setting to explore this because few large areas of continuous forest remain, with most of the habitat dominated by forest fragments [20]. Species richness is maximized in large areas of continuous forest, but many species still occupy forest fragments in several landscapes [21]. Because continuous and fragmented forests differ in the balance of solar radiation, temperature regime, nutrient cycling and hydrologic cycle [22] and in the microbial community structure of the environment [23], we focused our research on differences in the skin microbiota across these two forest types. Specifically, we studied the potential intrinsic lineage-specific influence on the skin microbiota of anurans by comparing sympatric species of amphibians native to the Atlantic Forest. The factor of fragmentation is likely a strong environmental influence on the skin microbiota in terms of diversity of bacteria. If the environmental conditions can exert some influence on the skin microbiota of amphibians, a symbiont community that offers protection against pathogens can be affected. That is why we explored and described the possible role of skin microbiota as antimicrobial agents by applying standard tests to a fraction of the bacterial forms that could be isolated from skin.

## Material and methods

### General approach

To test the hypothesis that bacterial communities of anuran skin vary among sympatric species of the Atlantic forest, we compared the composition of cutaneous microbiota in eight populations of four species of anurans from two forest types: fragments and continuous forest of South Eastern Atlantic Forest. Two continuous forests and associated fragments close enough to share basic climatic and physiognomic traits (see Study Areas) were compared. The general procedure started with field collection and identification of individual frogs followed by sampling of skin microbiota and estimation of the density and richness of bacterial colony forming units. Samples were then transported to the laboratory to isolate and identify bacterial strains based on sequence analysis of the 16S rRNA gene. The antimicrobial activity of each isolated morphotype was then evaluated.

We sampled four frog species, including *Dendropsophus minutus*, *Aplastodiscus leucopygius* and *Phyllomedusa distincta* (family Hylidae) and *Proceratophrys boiei* (family Odontophrynidae) (see Table 1). From the bacterial morphotypes isolated, we subsampled morphotypes for taxonomic identification, prioritizing those that exhibited antimicrobial effects and, as a secondary criterion, maximized morphological diversity in each host-species and sampling site.

**Table 1:** Total number of individuals of amphibians sampled, listed by species and sampling site.

Species	Continuous forests		Fragmented forests			
	INT	USV	RG.1	RG.2	SLP.1	SLP.2
*D*. *minutus*	17	34	-	16	-	7
*P*. *boiei*	17	5	12	-	18	-
*A*. *leucopygius*	-	14	-	-	14	-
*P*. *distincta*	16	-	18	-	-	-

**Abbreviations:** INT, Paraíso Eco Lodge farm; USV, Serra do Mar State Park–Unit of Santa Virgínia; RG, Ribeirão Grade municipality; SLP, São Luís do Paraitinga municipality.

### Study area

**Fig 1:**
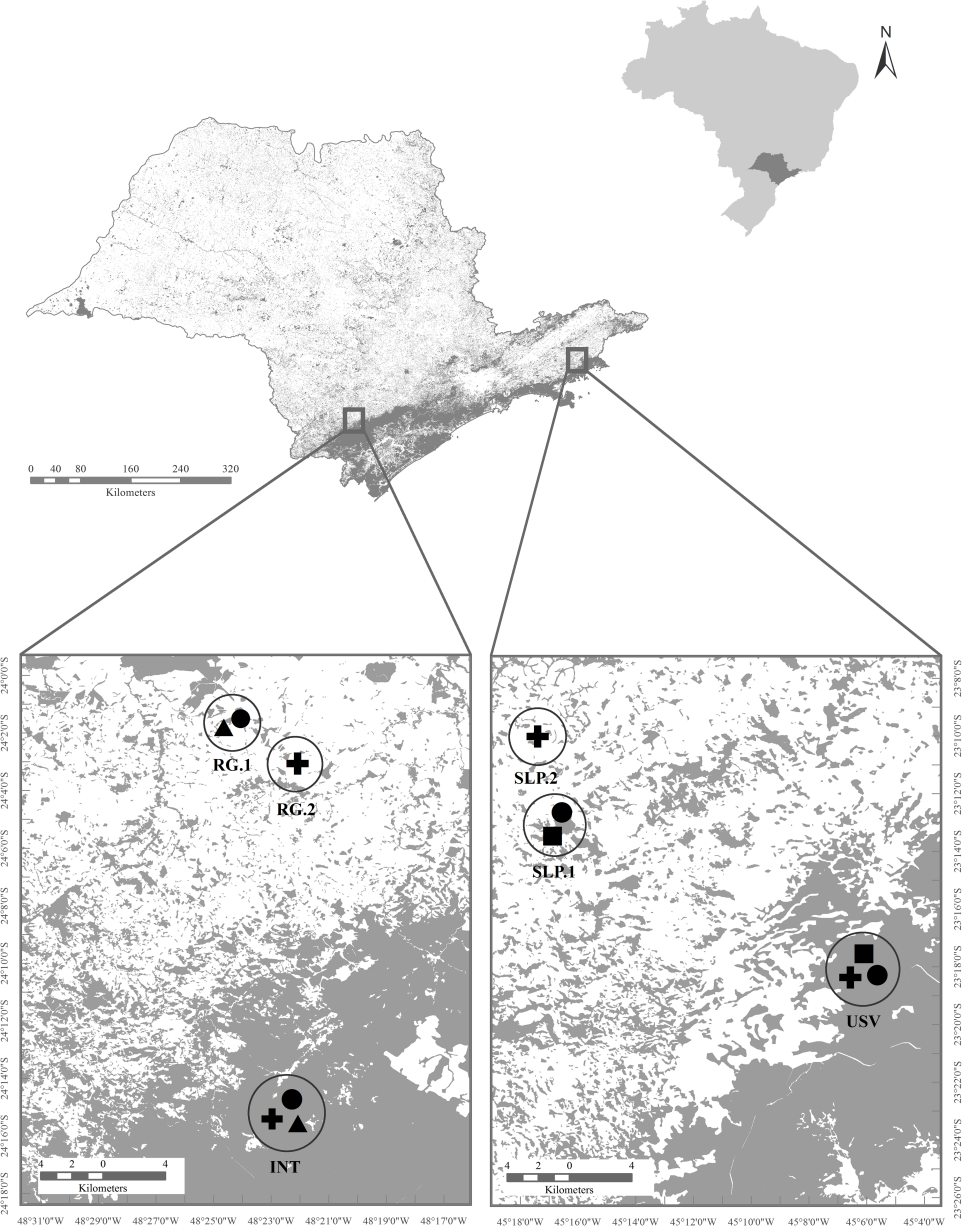
(A-B) Location of study area in the state of São Paulo with the native vegetation (grey areas) categorized according to the Forest Inventory of São Paulo [25]; (B) Sampling sites at Ribeirão Grande (RG) municipality in fragmented and continuous forests of the Paraíso Eco Lodge farm (INT); (C) Sampling sites at São Luís do Paraitinga (SLP) municipality in fragmented and continuous forests of Serra do Mar State Park–Unit of Santa Virgínia (USV). Each marked area refers to a single sampling site. Cross = *D*. *minutus*; Cicle = *P*. *boiei*; Triangle = *P*. *distincta*; Square = *A*. *leucopygius*.

**Fig 2:**
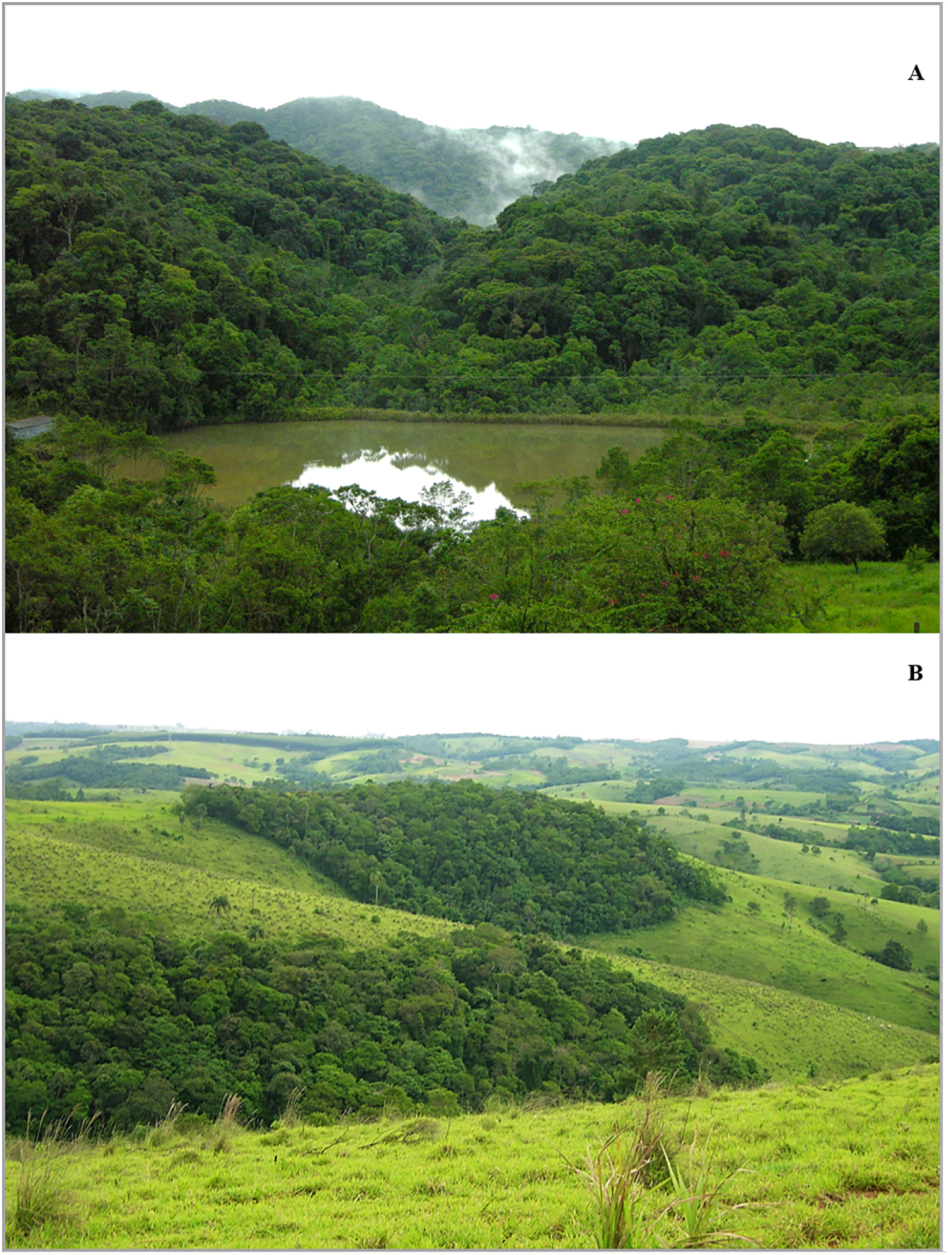
Photos illustrating the forest types studied. (A) Continuous forest of Atlantic Forest; (B) Fragmented forest of Atlantic Forest.

Our study was conducted in the State of São Paulo, Brazil, in contrasting areas of the Atlantic Forest: “*continuous forest*” (areas with significant extensions of protected forest) and “*fragments*” (patches of forested habitat with evident disruption of the unity of the landscape [24]). One continuous forest was the Serra do Mar State Park–Unit of Santa Virgínia (USV: 23°20'S, 45°08 W), which has an area of 175 km^2^, and 60% of primary forest (Forestry Foundation–Department of Environment of the State of São Paulo). Another continuous forest area was Paraíso Eco Lodge farm (INT: 24°14'S, 48°22'W), a locality within Intervales State Park in a forested area of 417, 04 km^2^, 60% considered primary forest (Forestry Foundation–Department of Environment of the State of São Paulo). Selected fragments were located at the municipalities of São Luís do Paraitinga (SLP: 23°13'S, 45°20'W) and Ribeirão Grande (RG: 24°05'S, 48°21'W), each with two fragments (Fig 1). The fragmented habitats were surrounded by a matrix dominated by pastures and monocultures (Fig 2). Santa Virgínia is 20 km from the fragments of São Luís do Paraitinga, about the same distance separating Eco Lodge from the fragments of Ribeirão Grande. The continuous forests are about 342 km apart.

### Sampling

We located and captured individual frogs at night during the reproductive period, November 2008 to March 2009, and October 2009 to December 2009, using active-search methods. In the fragmented areas of Ribeirão Grande, we captured frogs at the edge of the forests (*P*. *distincta*), temporary streams (*P*. *boiei*), and temporary ponds (*D*. *minutus*). In São Luís do Paraitinga fragments, we collected frogs along the courses of a permanent stream (*P*. *boiei*), a walking trail (*A*. *leucopygius*), and a temporary pond (*D*. *minutus*). In continuous forests, we collected frogs along trails in the Santa Virgínia section (*P*. *boiei* and *A*. *leucopygius*), at Paraíso Eco Lodge (*P*. *boiei* and *P*. *distincta)*, and at permanent ponds scattered in both localities (*D*. *minutus)*. Permission for amphibian sampling was obtained from the Brazilian Institute of Environment and Renewable Natural Resources (IBAMA; license number 16440–1) and the Forestry Institute of the state of São Paulo (SMA n° 260108–000.000.001.897/02008). All procedures were approved by the ethics committee for the use of animals in scientific experiments from the Biosciences Institute at the University of São Paulo (073/2008).

### Sampling and culturing the cutaneous microbiota

We captured frogs using sterile gloves disinfected with 70% alcohol, and used fresh gloves for each individual handled. Within 2h of capture, we washed frogs with distilled water to remove transient bacteria [26], then sampled the microbiota with a sterile swab. We swiped the entire length of the dorsum and venter, including head and throat region of the animal. After sampling, animals were returned to the place of capture. Swabs were placed individually into vials with 1ml of physiological solution (0.9% NaCl). Back at the lab, these were vigorously vortexed to release cells, then serially diluted to 1 x 10^−2^. We placed 100μl of the final dilution on R2A agar (Difco) plates for growth of heterotrophic bacteria and dispersed the sample using the spread plating technique. The dilution allowed the growth of 30 to 300 colonies per plate [27], and each sample was plated in triplicate.

Plates were incubated for 48 hours at room temperature, then the total number of colonies and morphotypes were counted after 48 and 72 hours of growth given that different species of bacteria exhibit different growth dynamics. Finally, three frogs of each species and locality were preserved into reference collection of our laboratory.

### Bacterial analysis

We used Heterotrophic Plating Count (HPC) to estimate the richness of bacterial morphotypes and the density of bacterial colonies. We recorded the total density of colonies as colony forming units (CFU/mL) [16], and used the mean of the three plates for the analysis. Variation in the bacterial density was not homogeneous among samples (Levene’s test, *P* < 0.05), so we used Welch’s ANOVA, followed by Games Howell post hoc test for pairwise comparison of species. We used ANOVA with randomization to compare forest type and sampling sites by species. Analysis of richness was assessed using G-tests because the cumulative number of morphotypes for each species, forest type and sampling site were not normally distributed.

### Isolation of bacterial morphotypes

We isolated and cultured all representative bacterial colonies from each sampling site and frog species. Each representative colony was called morphotype (i.e. colonies with the same morphological characteristics). We evaluated colony features such as color, shape, margin, elevation, brightness, size, surface (smooth or rough), and presence of granules using stereomicroscopy (Nikon SMZ 800). We transferred each morphotype isolated to fresh R2A culture medium and later stored these in glass tubes at 8°C.

### Identification of bacterial isolates

We identified the isolates by sequencing the 16S rRNA gene, obtained from amplified product with the primers 338F (5’-ACTCCTACGGGAGGCAGCAG-3’) and W031R (5’-TTACCGCGGCTGCTGGCAC-3’) [28]. The PCR reaction contained *Taq* DNA polymerase buffer, 0.25 μM dNTPs, 0.25 μM of each primer, 1.5 mM MgCl_2_, and 1.5 U Taq DNA polymerase. DNA templates were collected directly from the plate using a sterile toothpick and placed in the reaction solution (30 μL). The thermocycling parameters were 5 min at 94°C, followed by 30 cycles of 1 min at 94°C, 1 min at 61°C, 30 secs at 72°C, and 10 min at 72°C for the final elongation. We verified the amplification results by electrophoresis in 2% agarose gel, with ethidium bromide (0.5μg/ml) staining.

PCR products were cloned into the vector pGEM^®^ -T Easy (Promega), following the instructions provided by the manufacturer. Competent cells were transformed by electrical shock, following the Sambrook and colleagues [29] protocol. *E*. *coli* cells of the lineage SURE (Stratagene) exhibit the blue-white color for screening. Recombinant plasmid DNA extraction was obtained using a protocol from Birnboim and Doly [30]. DNA sequencing of the inserted PCR fragment was performed at the Institute of Chemistry, at the University of São Paulo–IQUSP and at the Universidade Católica de Brasilia, Brazil. The sequences were edited with the software BioEdit [31] and we used a phenetic analysis to compare the sequences obtained with those deposited in the database of the National Center for Biotechnology Information–NCBI–(2010) and Ribosomal Database Project, release 10—RDP-X using the Classifier and Sequence match tools [32]. The sequence length varied between 132 to 500 base pairs.

### Bacterial growth inhibition assay

To identify bacterial strains producing antibiotics, we used a modified cross-streak method originally described by Williston and colleagues [33]. Briefly, each isolate was inoculated on one half of a petri dish containing R2A medium for 48 hours or until growth was observed in the area inoculated. After this period, reference strains of pathogenic bacteria were inoculated in a perpendicular streak. Growth of these strains was evaluated after 24h at room temperature. The absence of growth on the perpendicular streaks indicated the antibiotic activity exhibited by the isolated bacteria.

We used this antimicrobial assay for bacterial species involved in amphibian diseases [34–40]. The reference strains were donated by the Oswaldo-Cruz Foundation–FIOCRUZ (RJ), and included the following: Gram-positive cocci *Staphylococcus aureus* –ATCC 14458; *Staphylococcus epidermidis*–ATCC 12228 and *Micrococcus luteus*–ATCC 7468, Bacilli Gram-negative: *Escherichia coli–* ATCC 11229; P*roteus vulgaris*–CCUG 10784; *Salmonella enterica subsp*. *enterica serovar Typhi* – ATCC 6539; *Salmonella enterica subsp*. *enterica serovar Enteritidis* (CT) –ATCC 13076; *Aeromonas hydrophila* –IOC/FDA 110–36; *Pseudomonas aeruginosa* –ATCC 15442 and *Klebsiella pneumoniae subsp*. *Pneumoniae–*ATCC 4352.

## Results

### Interspecific differences in the skin microbiota

There was no correlation between frog body size (snout-vent length) and the density of bacteria (CFU/mL) for three species: *A*. *leucopygius* (Spearman; ρ = -0.005, *P* = 0.978), *P*. *boiei* (Spearman; ρ = -0.122, *P* = 0.385), *P*. *distincta* (Spearman; ρ = -0.156, *P* = 0.378). We did find a correlation between body size and bacterial density in *D*. *minutus* (Spearman; ρ = 0.274, *P* = 0.019), but this is likely an idiosyncratic relationship in this species (*D*. *minutus* often did not generate colonies so that the data set presents several zero values. The relationship between bacterial density and body size is not monotonic and disappears if zero values are excluded). Body size was therefore not considered as a covariate in further analysis.

**Fig 3:**
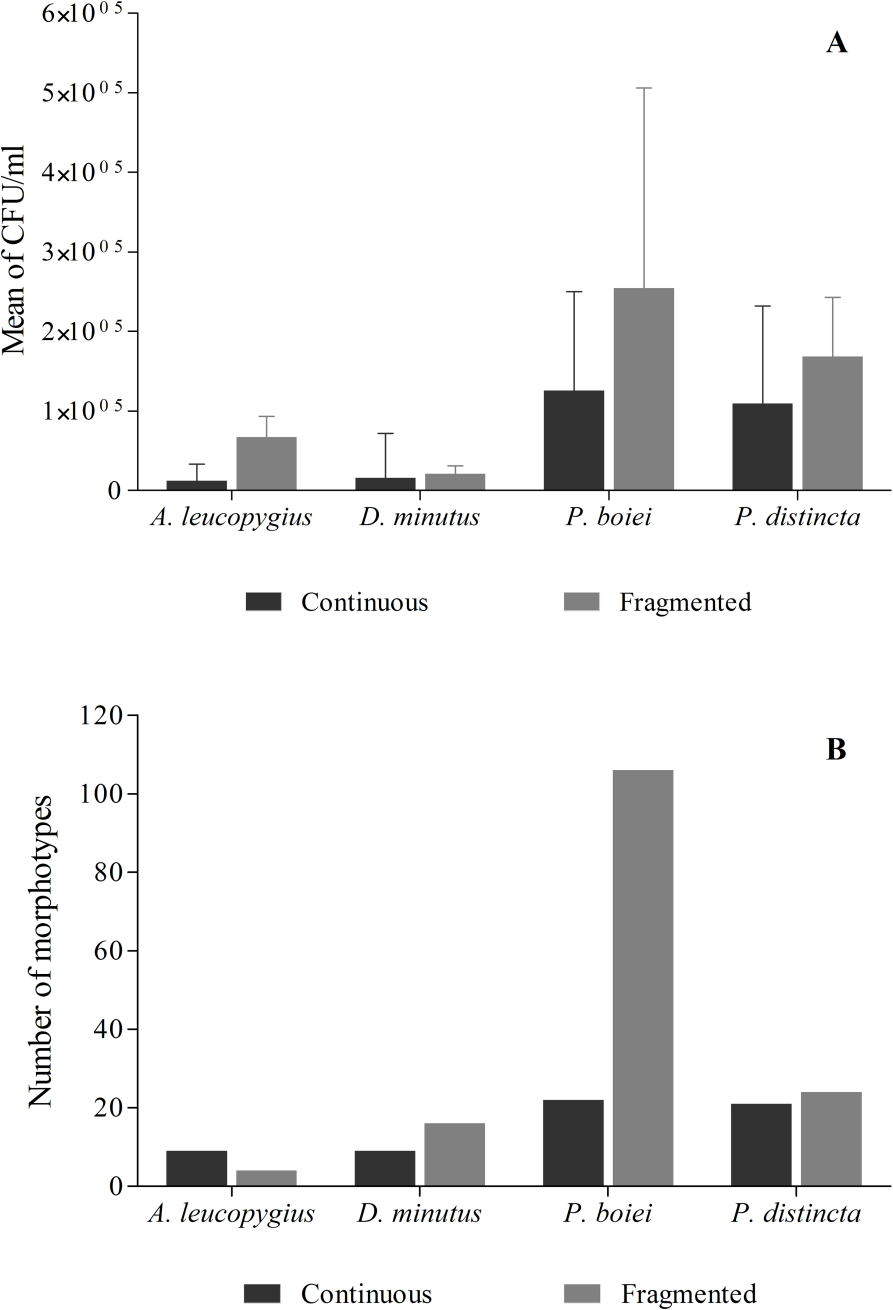
Comparisons of the mean density–CFU/mL–(3A) and total richness (3B) of bacterial morphotype colonies in the anuran species *P*. *boiei*, *P*. *distincta*, *A*. *leucopygius* and *D*. *minutus*, from continuous and fragmented forests.

Regardless of the forest type and sampling site, the microbiota of the amphibian species studied differed in density of CFUs (Welch’s Anova; F = 66.5, *P* < 0.0001, see Table 2) and in richness of bacterial morphotypes (G = 137.4, *P* < 0.001). The highest values of bacterial density and richness were observed on *P*. *boiei*, whereas *D*. *minutus* and *A*. *leucopygius* exhibited lower values (Fig 3). Some of the data on *P*. *distincta* were published previously by Assis and colleagues [41].

**Table 2:** Pairwise post-hoc comparisons (Games-Howell) and mean bacterial density (CFU/mL) of the amphibian species. Mean density values are Box-Cox transformation.

Species	Mean	SD	Post-hoc*
*P*. *boiei*	25,497	4,715	a
*P*. *distincta*	21,085	5,809	b
*D*. *minutus*	10,974	7,515	c
*A*. *leucopygius*	13,389	6,804	c

*Different letters indicate significant differences (p < 0.05) among species. For example, D minutus (c) is different than P. boiei (a) and P. distincta (b), but similar to A. leucopygius (c).

### Influence of the forest type on the skin microbiota

Because we observed interspecific differences in microbial parameters, we assessed how forest type influenced the microbiota by comparing density of CFUs and richness of morphotypes across individuals of each species. Individuals of *P*. *distincta* (Welch’s ANOVA; F = 0.908, *P* = 0.422), *A*. *leucopygius* (Welch’s ANOVA; F = 0.609, *P* = 0.639) and *D*. *minutus* (Welch’s ANOVA; F = 0.188, *P* = 0.779) displayed similar trends across the fragments and continuous forests not showing differences in the bacterial density between these forest type. The analysis revealed that only individuals of *P*. *boiei* (Welch’s ANOVA; F = 5.37, *P* = 0.013) exhibited differences in the bacterial density between individuals from fragments and continuous forest. The total number of cultivated morphotypes was two times greater in the forest fragments (149 morphotypes) compared with continuous forests (61 morphotypes). However, differences in the bacterial richness between these forests was observed only for *P*. *boiei* (G = 59.9, *P* < 0.001). Individuals of *P*. *distincta* (G = 0.22, *P* = 0.99), *D*. *minutus* (G = 1.40, *P* = 0.997) and *A*. *leucopygius* (G = 1.97, *P* = 0.991) exhibited similar values of bacterial richness across forest types (Fig 3).

### Differences between sampling sites

Variation in microbiota attributable to sampling site was assessed in *D*. *minutus* and *P*. *boiei* because only for these two species we had data comparing two sites within each forest type (continuous versus fragmented, see Fig 1). Variation in bacterial density among individuals of *D*. *minutus* was not influenced by sampling site in fragmented forests (Welch’s ANOVA; F = 0.166, *P* = 0.75), but sampling site was a significant factor for individuals from continuous forest (Welch’s ANOVA; F = 4.829, *P* = 0.004). Variation in bacterial density among individual *P*. *boiei* was not influenced by sampling site for either continuous (Welch’s ANOVA; F = 0.002, *P* = 0.958) or fragmented forests (Welch’s ANOVA; F = 0.822, *P* = 0.430). Morphotype richness was not significantly different between sampling sites for individuals of *D*. *minutus* from fragments (G = 14.7, *P* = 0.099) or continuous forests (G = 7.36, *P* = 0.599). There also were no significant differences in microbiota richness between sites for *P*. *boiei* from fragments (G = 3.07, *P* = 0.961) or from continuous forests (G = 0.18, *P* = 0.99).

### Taxonomic diversity

We identified 31 bacterial taxa belonging to various systematic levels (Table 3) from amphibians in fragmented forests, and 20 from equivalent samples in continuous forests. Only 13 of 29 total taxa occurred on frogs from both forest type. Only four phyla were observed: Proteobacteria (61%), Actinobacteria (22%), Firmicutes (11%) and Bacteroidetes (6%).

**Table 3:** Composition of bacterial species isolated from the skin of four anuran species and their occurrence.

Bacterial taxa	*P*. *boiei*	*P*. *distincta*	*A*. *leucopygius*	*D*. *minutus*	GenBank accession numbers*
FIRMICUTES	** **	** **	** **	** **	
Bacillaceae					
***Bacillus* sp.**	C	F	* *	F/C	**KY213902**/KY213897 KY213898/KY213899 KY213904/KY213910 KY213917/KP708598
Staphylococcaceae					
*Staphylococcus* sp.	* *	* *	F	* *	KY213945
BACTEROIDETES	** **	** **	** **	** **	
Sphingobacteriaceae					
*Pedobacter* sp.	F	F	C	* *	KY213903/KP708597 KY213926
Flavobacteriaceae					
***Flavobacterium* sp.**	F/C	*** ***	*** ***	*** ***	**KY213932**/KY213891 KY213892/KY213893
Bacteroidetes	F	* *	* *	* *	KY213941
ACTINOBACTERIA	** **	** **	** **	** **	
Microbacteriaceae					
**Microbacteriaceae**	F/C	F	*** ***	F	KY213908 /KY213942 KY213905/KY213915
*Microbacterium* sp.		F	*** ***		KP708605
*Curtobacterium* sp.		F	*** ***		KP708600
Nocardiaceae					
*Nocardioides* sp.	F	* *	* *	* *	KY213922
Patulibacteriaceae					
*Patulibacter* sp.	* *	F	* *	* *	KP708599/KP708606
Streptomycetaceae	* *		* *	* *	
***Streptomyces* sp.**	F	** **	*** ***	*** ***	**KY213929/KY213947**
Actinomycetales	F		C	* *	KY213943/KY213912 KY213934/KY213911
PROTEOBACTERIA	** **	** **	** **	** **	
Xanthomonadaceae					
**Xanthomonadaceae**	F	* *	* *	* *	**KY213927**/KY213920
***Lysobacter* sp.**	F	* *	* *	* *	**KY213948**
***Stenotrophomonas* sp.**	F/C	*** ***	*** ***	*** ***	**KY213936**/KY213895
***Luteimonas* sp.**	F	*** ***	*** ***	*** ***	**KY213930**
Enterobacteriaceae					
**Enterobacteriaceae**	F/C	F/C	F		**KY213921**/KY213925 KY213937/KY213938 KX632217/KP708589 KP708590/KX632214 KP708583/KP708588 KY213906
*Pantoea* sp.		F			KP708601/KP708602
***Enterobacter* sp.**		C			**KP708587**/KP708596
*Escherichia* sp	* *	* *	F	* *	KY213939
***Serratia* sp.**	*** ***	C	*** ***	*** ***	**KP708585/**KP708585 KP708591
Citrobacter*	* *	C	* *	* *	KP708595
Erwinia*	* *	F/C	* *	* *	KP708593/KP708594 KP708604
Pseudomonadaceae					
**Pseudomonadaceae**	F/C		F	* *	**KY213924**/KY213935 KY213894/KY213940
***Pseudomonas* sp.**	C	C	C	F	**KP708586/KY213913** **KY213914/KY213916** **KY213949**/**KY213928** KP708584/KP708592 KY213896
Comamonadaceae					
**Comamonadaceae**	F/C	** **	** **	** **	**KY213944/KY213907**
Moraxellaceae					
***Acinetobacter* sp.**	F	F	F	* *	**KY213931**/**KY213946** **KP708603**/KY213933
Oxalobacteraceae				* *	
Oxalobacteraceae				C	KY213901
Burkholderiales				C	KY213900
Sphingomonadales	F				KY213919
Rhizobiales	C/F	*** ***	*** ***	*** ***	KY213909/KY213923
**Gammaproteobacteria**	F	*** ***	*** ***	*** ***	**KY213918**

**Abbreviations:** F, fragmented landscape; C, continuous landscape; Bold (except Phylum): bacteria with antimicrobial activity. For each isolated bacterium. **Obs**.: Data on *P*. *distincta* were partially published previously by Assis and colleagues [41].

Composition of bacterial communities was different depending on the amphibian species. We identified nineteen taxa of bacteria isolated from *P*. *boiei*, ten taxa from *A*. *leucopygius*, nine taxa from *P*. *distincta* and six taxa from *D*. *minutus*. Only bacteria belonging to the genus *Pseudomonas* was common to all studied species. *Proceratophrys boiei* had the highest richness of bacterial morphotypes and the greatest proportion of exclusive taxa (37.9%). Bacteria from the genera *Stenotrophomonas*, *Streptomyces*, *Lysobacter*, *Nocardioides*, *Flavobacterium* and the families Comamonadaceae, Flavobacteriaceae, Nocardioidaceae, Streptomycetaceae and Xanthomonadaceae were detected only on *P*. *boiei*. In *A*. *leucopygius*, bacteria from the genera *Escherichia*, *Patulibacter*, *Staphylococcus* and *Microbacterium* were represented, as well as the families Patulibacteraceae and Staphylococcaceae. Bacteria from the genera *Citrobacter*, *Erwinia*, *Serratia* and *Enterobacter* were isolated from individuals of *P*. *distincta*, and bacteria from the order Burkholderiales and the family Oxalobacteraceae were found exclusively on *D*. *minutus* (Table 3).

### Antimicrobial activity

From the 221 isolated bacterial morphotypes, 27 showed antimicrobial activity against at least one of the 10 pathogens tested. Of these 27, we identified 24 morphotypes using molecular methods (Table 4). *Proceratophrys boiei* had the greatest number of inhibitory morphotypes (17), followed by *P*. *distincta* (4), *D*. *minutus* (3), and *A*. *leucopygius* (3). The ratio of the number of isolated morphotypes to the number of antimicrobial morphotypes was 4:1 for *A*. *leucopygius*, 7:1 for *P*. *boiei*, 8:1 for *D*. *minutus*, and 11:1 for *P*. *distincta*. There were also differences in the antimicrobial activity among the bacterial strains isolated. One strain of the genus *Streptomyces*, isolated from *P*. *boiei*, exhibited the strongest antimicrobial activity, inhibiting all the pathogens tested. Skin microbes from frogs were highly effective against *S*. *aureus*, which was the most inhibited reference strain and exhibited growth inhibition by 16 isolated bacteria. Other pathogenic bacteria exhibiting growth inhibition were *A*. *hydrophila* (11 strains), *M*. *luteus* (11), *K*. *pneumoniae* (8), *P*. *vulgaris* (5), *S*. *typhi* (4), *E*. *coli* (3), *P*. *aeruginosa* (3), and *S*. *enteritidis* (1) (see Table 4).

**Table 4:** Inhibitory effect of bacterial strain from amphibian species, by each tested pathogen.

Species	Morphotypes	EC	AH	PA	SA	SE	ST	SEP	PV	ML	KP	GenBank accession numbers
*P*. *boiei*	*Streptomyces* sp.	*	*	*	*	*	*	*	*	*	*	KY213947
	*Flavobacterium* sp.	*	-	*	*	-	*	-	-	*	*	KY213932
	Xanthomonadaceae	-	*	-	*	-	-	*	-	*	-	KY213927
	Pseudomonadaceae	*	*	-	*	-	-	*	-	-	-	KY213924
	*Acinetobacter* sp.	-	-	-	-	-	-	*	*	*	*	KY213931
	Comamonadaceae	-	-	-	-	-	-	-	*	*	*	KY213944
	Gammaproteobacteria	-	-	-	*	-	-	-	*	-	*	KY213918
	*Pseudomonas* sp.	-	-	-	*	-	-	*	-	*		KY213949
	USV95-167**	-	*	-	*	-	-	*	-	-	-	
	Comamonadaceae	-	-	-	-	-	-	*	-	*	-	KY213907
	USV95-166**	-	-	-	-	-	-	*	-	-	*	
	*Streptomyces* sp.	-	*	-	*	-	-	-	-	-	-	KY213929
	Enterobacteriaceae	-	*	-	-	-	-	-	-	-	-	KY213921
	*Stenotrophomonas* sp.	-	-	-	-	-	-	*	-	-	-	KY213930
	INT70-148**	-	-	-	*	-	-	-	-	-	-	
	*Lysobacter* sp.	-	-	-	-	-	-	*	-	-	-	KY213948
	*Stenotrophomonas* sp.	-	-	-	-	-	-	*	-	-	-	KY213936
*P*.*distincta*	*Serratia* sp.	-	-	-	*	-	*	-	-	-	-	KP708585
	*Pseudomonas* sp.	-	*	-	*	-	-	-	-	-	-	KP708586
	*Enterobacter* sp.	-	-	-	*	-	-	-	-	-	-	KP708587
	*Acinetobacter* sp.	-	-	-	-	-	-	*	-	-	-	KP708603
*A*.*leucopygius*	*Pseudomonas* sp.	-	*	-	*	-	-	*	-	*	*	KY213914
	*Pseudomonas* sp.	-	*	-	*	-	-	*	-	*	-	KY213913
	*Acinetobacter* sp.	-	-	-	-	-	-	*	-	-	-	KY213946
*D*.*minutus*	*Pseudomonas* sp.	-	*	*	*	-	-	*	*	*	*	KY213916
	*Pseudomonas* sp.	-	*	-	*	-	-	*	-	*	-	KY213928
	*Bacillus* sp.	-	-	-	-	-	*	-	-	-	-	KY213902

**Abbreviations**: EC, *Escherichia coli;* AH, *Aeromonas hydrophila;* PA, *Pseudomonas aeruginosa;* SA, *Staphyllococcus aureus;* SE, *Streptococcus epidermidis;* ST, *Salmonella typhi;* SEP, *Streptococcus epidermidis;* PV, *Proteus vulgaris;* ML, *Micrococcus luteus;* KP, *Klebisiella pneumoniae*.

* indicate the pathogen inhibition was observed.

** DNA sequence not obtained. **Obs**.: Data on *P*. *distincta* were partially published previously by Assis and colleagues [41].

## Discussion

Our measures are based on culturable bacterial diversity, which is a partial, yet expressive representation of the cutaneous microbiota of the anuran community, as showed by Walke and colleagues [42]. We conducted this research under the perspective that the anuran skin microbiota is influenced by intrinsic factors, and we expected that sympatric species would differ, and our data on bacterial density and richness support this view. Environmental influences exist, but those are also species-specific in a way that corroborates the hypothesis of specificity of the amphibian skin microbiota. Our results add to those of McKenzie and colleagues [43], who observed differences in the community composition of the skin microbiota among the frog species *Lithobates pipiens*, *Pseudacris triseriata* and the salamander *Ambystoma tigrinum*, Belden and colleagues [44], who described differences in the skin microbiota of the frog species *Agalychnis callidryas*, *Dendropsophus ebraccatus* and *Craugastor fitzinger*, based on operational taxonomic unit and metabolite profiles, and Kueneman and colleagues [45], who showed that host species predicts the skin microbial community composition in the species *Anaxyrus boreas*, *Pseudacris regilla* and *Lithobates catesbeianus*, and the salamander *Taricha torosa*. The latter research also showed an influence of the sampling site on the skin microbiota, though the authors considered this factor as secondary in assessing the composition of these microbial communities. In the current study, we found different effects of the sampling sites for the studied species. Once a strong effect of the species on the skin microbiota was found, an intrinsic influence, as variation in the genetic population, could further explain the variation in the skin bacterial density in *D*. *minutus*. The overall body of literature suggests lineage-specific traits, but we believe that more information is necessary to propose generalizations and fully understand factors that influence the structure and function of the amphibian skin microbiota. Woodhams and colleagues [46] have established a database with 16S rRNA gene sequences of amphibian skin-associated bacteria that exhibit anti-microbial activity. This is a promising endeavor, although this database is restricted to bacterial isolates that presented antimicrobial activity only against the chytrid fungus (*Bd*).

Our results indicate an environmental correlation on the structure of the amphibian skin microbiota, but we cannot evaluate its functional significance or underlying causative mechanisms. Physiological responses to environment and natural history greatly influence skin microbiota through the host’s behavior and ecology. The species with greatest bacterial diversity and density was *P*. *boiei*, the only species in our study that lives in leaf litter. This relationship is not surprising given that individual frogs of this species are likely exposed to the highest levels of microbial diversity. In contrast, the lowest microbial variation occurred in *D*. *minutus*, which is strongly associated with water and likely experiences frequent washing. Also, properties of skin secretions may serve as a negative filter (to determine viable bacterial groups) because of their composition and profile as antimicrobial bioactive molecules [19]. However, positive filters may also apply because bacteria differ in preferred nutritional substrates. For example, bacteria of the family Xanthomonadaceae, whose members were found only on *P*. *boiei*, use a restricted spectrum of nutritive sources [47]. In contrast, bacteria of Pseudomonadaceae, regarded as nutritional generalists [48], occur in the microbiota of all studied species. Bacterial communities on frog skin may be affected by the properties of the mucous, secreted on the amphibian skin, which is rich in glycoproteins and can be used for the bacterial nutrition [17].

Recent research on the frog skin microbiota diversity relies on molecular data that allow the investigation of uncultured bacteria species, as shown above. However, in the present work, the analysis of culturable bacteria allowed further investigation of the antimicrobial potential of the skin microbiota. Different ratios of the number of isolated bacteria and the number of isolated bacteria with antimicrobial activity suggest interspecific differences in the potential of the skin microbiota against the colonization by pathogenic microorganisms. Rebollar and colleagues [49] found similarities between skin bacterial communities of amphibian species based on susceptibility to the chytrid fungus *Bd*. We found that the greatest number of morphotypes with inhibitory power occur in *P*. *boiei*, which may reflect that the richest bacterial community occurs on a frog that lives in leaf litter and is exposed to soil bacterial species, many of which are known to produce antibiotics [50]. The identified families of bacteria include those that produce strong antimicrobial substances such as Xanthomonadaceae [51,52], Flavobacteriaceae [53] and Bacillaceae [54]. The antibacterial genera *Pseudomonas*, *Acinetobacter*, *Stenotrophomonas* and *Comamonas* (family Comamonadacea as identified in this study) were previously detected on frogs of the genus *Atelopus*. Some members of these genera are effective in inhibiting growth of the fungus *Bd* [55]. This overlap of antimicrobial bacteria on frogs that are phylogenetically distant could indicate a selective process in the amphibian skin, promoted by intrinsic characteristics of the group. A key point, however, is that no direct inference to field conditions can be made from laboratory tests with isolated bacterial morphotypes. In vivo, these morphotypes may act as communities, possibly with emergent properties and modulation that cannot be addressed in our study.

In summary, results of this study support the hypothesis that bacterial communities on the amphibian skin are lineage specific, but may be influenced by environmental factors depending on the frog species given that some have protective properties of the skin. A complex network of factors composes the “dermosphere”, where intrinsic properties of the skin integrate with external factors. The interaction between intrinsic and external properties overall provides nutrients, a biochemical profile and a range microclimate conditions that influence the colonization of the skin.

## Supporting information

S1 File Quantitative summary of the research. (PDF)
