# Supplementary material for: Skin microbiota in frogs from the Brazilian Atlantic Forest: Species, forest type, and potential against pathogens
Source: PLoS One. 2017 Jul 5;12(7):e0179628. doi: 10.1371/journal.pone.0179628 (PMC5497969; doi:10.1371/journal.pone.0179628)
Supplement: S1 File — (PDF) [file pone.0179628.s001.pdf]

Individual	Species	Forest type	Sampling site	Bacterial density (CFU/mL)	Morphotypes
USV116	*A. leucopygius*	Continuous	USV	4,67E+03	0
USV117	*A. leucopygius*	Continuous	USV	1,00E+03	0
USV118	*A. leucopygius*	Continuous	USV	3,00E+03	0
USV160	*A. leucopygius*	Continuous	USV	6,53E+04	0
USV161	*A. leucopygius*	Continuous	USV	7,00E+03	0
USV162	*A. leucopygius*	Continuous	USV	0,00E+00	0
USV163	*A. leucopygius*	Continuous	USV	6,67E+02	0
USV164	*A. leucopygius*	Continuous	USV	8,33E+03	0
USV165	*A. leucopygius*	Continuous	USV	1,00E+03	0
USV85	*A. leucopygius*	Continuous	USV	2,67E+04	2
USV96	*A. leucopygius*	Continuous	USV	0,00E+00	0
USV97	*A. leucopygius*	Continuous	USV	1,00E+03	2
USV98	*A. leucopygius*	Continuous	USV	1,00E+03	2
USV99	*A. leucopygius*	Continuous	USV	5,07E+04	3
SLP120	*A. leucopygius*	Fragmented	SLP.1	2,67E+03	0
SLP121	*A. leucopygius*	Fragmented	SLP.1	3,33E+02	0
SLP122	*A. leucopygius*	Fragmented	SLP.1	6,67E+02	0
SLP123	*A. leucopygius*	Fragmented	SLP.1	2,33E+03	0
SLP146	*A. leucopygius*	Fragmented	SLP.1	0,00E+00	0
SLP147	*A. leucopygius*	Fragmented	SLP.1	3,33E+02	0
SLP154	*A. leucopygius*	Fragmented	SLP.1	3,00E+03	0
SLP155	*A. leucopygius*	Fragmented	SLP.1	4,27E+04	0
SLP156	*A. leucopygius*	Fragmented	SLP.1	4,50E+04	0
SLP157	*A. leucopygius*	Fragmented	SLP.1	1,47E+04	0
SLP158	*A. leucopygius*	Fragmented	SLP.1	2,67E+03	0
SLP159	*A. leucopygius*	Fragmented	SLP.1	3,67E+03	0
SLP100	*A. leucopygius*	Fragmented	SLP.1	2,45E+05	1
SLP16	*A. leucopygius*	Fragmented	SLP.1	6,00E+03	3
INT199	*D. minutus*	Continuous	INT	5,00E+02	0
INT200	*D. minutus*	Continuous	INT	1,67E+02	0
INT201	*D. minutus*	Continuous	INT	1,70E+03	0
INT202	*D. minutus*	Continuous	INT	7,30E+04	0
INT203	*D. minutus*	Continuous	INT	1,16E+05	0
INT204	*D. minutus*	Continuous	INT	2,63E+04	0
INT72	*D. minutus*	Continuous	INT	1,57E+04	0
INT73	*D. minutus*	Continuous	INT	1,10E+04	3
INT74	*D. minutus*	Continuous	INT	6,67E+02	1
INT75	*D. minutus*	Continuous	INT	5,33E+03	2
INT76	*D. minutus*	Continuous	INT	3,67E+03	3
INT77	*D. minutus*	Continuous	INT	3,33E+02	0
INT78	*D. minutus*	Continuous	INT	6,67E+02	0
INT79	*D. minutus*	Continuous	INT	9,00E+03	0

INT80	*D. minutus*	Continuous	INT	2,17E+04	0
INT81	*D. minutus*	Continuous	INT	3,75E+05	0
INT82	*D. minutus*	Continuous	INT	3,33E+03	0
USV105	*D. minutus*	Continuous	USV	0,00E+00	0
USV106	*D. minutus*	Continuous	USV	1,67E+03	0
USV 107	*D. minutus*	Continuous	USV	2,67E+03	0
USV108	*D. minutus*	Continuous	USV	2,67E+03	0
USV109	*D. minutus*	Continuous	USV	7,00E+03	0
USV110	*D. minutus*	Continuous	USV	4,67E+03	0
USV111	*D. minutus*	Continuous	USV	7,33E+03	0
USV112	*D. minutus*	Continuous	USV	5,67E+03	0
USV113	*D. minutus*	Continuous	USV	7,33E+03	0
USV114	*D. minutus*	Continuous	USV	1,33E+03	0
USV115	*D. minutus*	Continuous	USV	4,33E+03	0
USV136	*D. minutus*	Continuous	USV	6,67E+02	0
USV137	*D. minutus*	Continuous	USV	4,13E+04	0
USV138	*D. minutus*	Continuous	USV	6,67E+02	0
USV139	*D. minutus*	Continuous	USV	3,33E+02	0
USV140	*D. minutus*	Continuous	USV	6,67E+02	0
USV141	*D. minutus*	Continuous	USV	3,33E+02	0
USV142	*D. minutus*	Continuous	USV	3,33E+02	0
USV143	*D. minutus*	Continuous	USV	1,00E+03	0
USV144	*D. minutus*	Continuous	USV	3,57E+04	0
USV145	*D. minutus*	Continuous	USV	3,33E+02	0
USV171	*D. minutus*	Continuous	USV	1,00E+03	0
USV172	*D. minutus*	Continuous	USV	1,00E+03	0
USV83	*D. minutus*	Continuous	USV	0,00E+00	0
USV84	*D. minutus*	Continuous	USV	0,00E+00	0
USV86	*D. minutus*	Continuous	USV	0,00E+00	0
USV87	*D. minutus*	Continuous	USV	0,00E+00	0
USV88	*D. minutus*	Continuous	USV	3,33E+02	0
USV89	*D. minutus*	Continuous	USV	0,00E+00	0
USV90	*D. minutus*	Continuous	USV	0,00E+00	0
USV91	*D. minutus*	Continuous	USV	not included	1
USV92	*D. minutus*	Continuous	USV	0,00E+00	0
USV93	*D. minutus*	Continuous	USV	0,00E+00	0
USV94	*D. minutus*	Continuous	USV	1,40E+04	0
RG101	*D. minutus*	Fragmented	RG.2	3,93E+04	4
RG102	*D. minutus*	Fragmented	RG.2	7,00E+03	4
RG103	*D. minutus*	Fragmented	RG.2	4,77E+04	6
RG104	*D. minutus*	Fragmented	RG.2	3,33E+03	1
RG177	*D. minutus*	Fragmented	RG.2	2,00E+03	0
RG178	*D. minutus*	Fragmented	RG.2	1,97E+04	0
RG179	*D. minutus*	Fragmented	RG.2	4,00E+03	0
RG180	*D. minutus*	Fragmented	RG.2	1,33E+03	0
RG181	*D. minutus*	Fragmented	RG.2	5,33E+03	0

RG182	*D. minutus*	Fragmented	RG.2	0,00E+00	0
RG183	*D. minutus*	Fragmented	RG.2	0,00E+00	0
RG184	*D. minutus*	Fragmented	RG.2	3,33E+03	0
RG185	*D. minutus*	Fragmented	RG.2	3,33E+02	0
RG186	*D. minutus*	Fragmented	RG.2	3,00E+03	0
RG187	*D. minutus*	Fragmented	RG.2	6,67E+02	0
RG188	*D. minutus*	Fragmented	RG.2	0,00E+00	0
SLP132	*D. minutus*	Fragmented	SLP.2	0,00E+00	1
SLP133	*D. minutus*	Fragmented	SLP.2	0,00E+00	0
SLP134	*D. minutus*	Fragmented	SLP.2	0,00E+00	0
SLP135	*D. minutus*	Fragmented	SLP.2	0,00E+00	0
SLP173	*D. minutus*	Fragmented	SLP.2	8,77E+04	0
SLP175	*D. minutus*	Fragmented	SLP.2	6,00E+03	0
SLP176	*D. minutus*	Fragmented	SLP.2	2,00E+03	0
INT192	*P. boiei*	Continuous	INT	2,01E+04	0
INT193	*P. boiei*	Continuous	INT	9,17E+03	0
INT194	*P. boiei*	Continuous	INT	2,22E+04	0
INT195	*P. boiei*	Continuous	INT	6,77E+03	0
INT196	*P. boiei*	Continuous	INT	9,07E+03	0
INT197	*P. boiei*	Continuous	INT	2,30E+04	0
INT198	*P. boiei*	Continuous	INT	3,80E+04	0
INT33	*P. boiei*	Continuous	INT	2,73E+05	0
INT34	*P. boiei*	Continuous	INT	2,77E+05	1
INT35	*P. boiei*	Continuous	INT	2,00E+05	4
INT36	*P. boiei*	Continuous	INT	1,93E+04	0
INT37	*P. boiei*	Continuous	INT	1,20E+05	4
INT38	*P. boiei*	Continuous	INT	1,21E+05	0
INT39	*P. boiei*	Continuous	INT	1,29E+05	0
INT40	*P. boiei*	Continuous	INT	4,88E+05	1
INT70	*P. boiei*	Continuous	INT	2,43E+05	2
INT71	*P. boiei*	Continuous	INT	1,63E+04	0
USV166	*P. boiei*	Continuous	USV	6,70E+04	0
USV167	*P. boiei*	Continuous	USV	2,01E+05	0
USV168	*P. boiei*	Continuous	USV	6,73E+04	0
USV169	*P. boiei*	Continuous	USV	2,20E+05	0
USV95	*P. boiei*	Continuous	USV	1,93E+05	10
RG189	*P. boiei*	Fragmented	RG.1	2,31E+05	0
RG190	*P. boiei*	Fragmented	RG.1	1,14E+05	0
RG191	*P. boiei*	Fragmented	RG.1	1,50E+05	0
RG28	*P. boiei*	Fragmented	RG.1	3,18E+05	22
RG29	*P. boiei*	Fragmented	RG.1	1,90E+05	14
RG30	*P. boiei*	Fragmented	RG.1	2,88E+05	13
RG31	*P. boiei*	Fragmented	RG.1	1,46E+05	5
RG32	*P. boiei*	Fragmented	RG.1	1,37E+05	1
RG56	*P. boiei*	Fragmented	RG.1	2,11E+05	4
RG57	*P. boiei*	Fragmented	RG.1	2,01E+05	1

RG58	*P. boiei*	Fragmented	RG.1	2,41E+05	2
RG59	*P. boiei*	Fragmented	RG.1	2,55E+05	0
SLP124	*P. boiei*	Fragmented	SLP.1	1,95E+05	11
SLP125	*P. boiei*	Fragmented	SLP.1	1,95E+05	3
SLP126	*P. boiei*	Fragmented	SLP.1	1,88E+05	0
SLP127	*P. boiei*	Fragmented	SLP.1	2,18E+05	2
SLP128	*P. boiei*	Fragmented	SLP.1	1,46E+05	2
SLP129	*P. boiei*	Fragmented	SLP.1	3,89E+05	0
SLP130	*P. boiei*	Fragmented	SLP.1	1,42E+05	0
SLP131	*P. boiei*	Fragmented	SLP.1	3,50E+04	0
SLP148	*P. boiei*	Fragmented	SLP.1	1,54E+05	1
SLP149	*P. boiei*	Fragmented	SLP.1	1,28E+06	1
SLP150	*P. boiei*	Fragmented	SLP.1	3,23E+05	1
SLP151	*P. boiei*	Fragmented	SLP.1	9,93E+05	0
SLP152	*P. boiei*	Fragmented	SLP.1	1,13E+05	1
SLP1	*P. boiei*	Fragmented	SLP.1	1,68E+05	0
SLP2	*P. boiei*	Fragmented	SLP.1	6,85E+04	14
SLP3	*P. boiei*	Fragmented	SLP.1	1,67E+05	3
SLP4	*P. boiei*	Fragmented	SLP.1	1,54E+05	5
SLP5	*P. boiei*	Fragmented	SLP.1	1,45E+05	0
INT18	*P. distincta*	Continuous	INT	not included	not included
INT19	*P. distincta*	Continuous	INT	1,38E+05	1
INT20	*P. distincta*	Continuous	INT	1,00E+03	3
INT205	*P. distincta*	Continuous	INT	5,33E+03	0
INT206	*P. distincta*	Continuous	INT	1,37E+05	0
INT207	*P. distincta*	Continuous	INT	3,30E+04	0
INT208	*P. distincta*	Continuous	INT	1,14E+05	0
INT21	*P. distincta*	Continuous	INT	1,38E+05	0
INT22	*P. distincta*	Continuous	INT	8,75E+04	3
INT23	*P. distincta*	Continuous	INT	1,05E+05	2
INT24	*P. distincta*	Continuous	INT	9,95E+04	0
INT25	*P. distincta*	Continuous	INT	1,41E+05	0
INT26	*P. distincta*	Continuous	INT	2,50E+04	3
INT27	*P. distincta*	Continuous	INT	5,09E+05	3
INT68	*P. distincta*	Continuous	INT	1,08E+05	4
INT69	*P. distincta*	Continuous	INT	3,33E+03	2
RG46	*P. distincta*	Fragmented	RG.1	1,50E+04	0
RG47	*P. distincta*	Fragmented	RG.1	2,00E+03	0
RG48	*P. distincta*	Fragmented	RG.1	7,67E+03	0
RG49	*P. distincta*	Fragmented	RG.1	4,33E+03	0
RG50	*P. distincta*	Fragmented	RG.1	3,33E+02	0
RG51	*P. distincta*	Fragmented	RG.1	7,67E+03	0
RG52	*P. distincta*	Fragmented	RG.1	1,43E+04	0
RG53	*P. distincta*	Fragmented	RG.1	9,00E+03	4
RG54	*P. distincta*	Fragmented	RG.1	7,13E+04	0
RG55	*P. distincta*	Fragmented	RG.1	4,87E+04	12

RG60	*P. distincta*	Fragmented	RG.1	8,67E+04	0
RG61	*P. distincta*	Fragmented	RG.1	6,33E+04	2
RG62	*P. distincta*	Fragmented	RG.1	3,87E+04	0
RG63	*P. distincta*	Fragmented	RG.1	4,00E+04	1
RG64	*P. distincta*	Fragmented	RG.1	7,14E+05	1
RG65	*P. distincta*	Fragmented	RG.1	1,53E+04	2
RG66	*P. distincta*	Fragmented	RG.1	1,25E+05	2
RG67	*P. distincta*	Fragmented	RG.1	not included	not included

Abbreviations: USV, Unit of Santa Virgínia; SLP, São Luís do Paraitinga city; INT, Intervales - Paraíso farm; RG, Ribeirão Grande city. **Legend**: not included, samples which presented some problem during proceedings.
